# Circulating Zonulin in Irritable Bowel Syndrome: A Systematic Review of Conflicting Evidence and the Critical Impact of Assay Specificity

**DOI:** 10.3390/biomedicines14102354

**Published:** 2026-10-16

**Authors:** Iulia Cristina Marginean, Cristina Maria Marginean, Sergiu Marian Cazacu, George Alexandru Iacob, Dragos Fortofoiu, Marian Catalin Otoiu, Mihaela Popescu, Cristin Constantin Vere

**Affiliations:** 1 Doctoral School, University of Medicine and Pharmacy of Craiova, 200349 Craiova, Romania; iulia.cristina18@yahoo.com (I.C.M.); dragos.fortofoiu@umfcv.ro (D.F.); otoiucatalin@yahoo.com (M.C.O.); 2 Department of Internal Medicine, University of Medicine and Pharmacy of Craiova, 200349 Craiova, Romania; 3 Research Center of Gastroenterology and Hepatology, University of Medicine and Pharmacy of Craiova, 200349 Craiova, Romania; vere_cristin@yahoo.com; 4 Department of Radiology and Medical Imaging, Clinical Emergency County Hospital Craiova, 200349 Craiova, Romania; georgeicb5@gmail.com; 5 Department of Endocrinology, University of Medicine and Pharmacy of Craiova, 200349 Craiova, Romania; mihaela.n.popescu99@gmail.com

**Keywords:** zonulin, irritable bowel syndrome, intestinal permeability, biomarker, ELISA, pre-haptoglobin-2

## Abstract

**Background:** Increased intestinal permeability is a proposed pathophysiological mechanism in irritable bowel syndrome (IBS), and zonulin, a putative regulator of tight junctions, has been investigated as a non-invasive serum biomarker. However, the diagnostic utility of circulating zonulin in IBS remains controversial. **Objectives**: To systematically review studies comparing serum or plasma zonulin concentrations between adult IBS patients and healthy controls, and to evaluate the influence of assay methodology on reported outcomes. **Methods**: A systematic literature search of PubMed was performed up to April 2026; the most recent eligible study was published in June 2024. Studies that enrolled adults with IBS diagnosed by Rome criteria, included healthy control groups, and measured zonulin by enzyme-linked immunosorbent assay (ELISA) were eligible. Studies lacking a healthy control group, non-original reports, animal studies, and mixed functional gastrointestinal disorder cohorts without extractable IBS data were excluded. Findings were synthesised narratively due to substantial methodological heterogeneity and concerns regarding the validity of commercially available zonulin kits. Risk of bias was assessed using the Newcastle–Ottawa Scale for case–control studies; six studies were at low risk and two at intermediate risk. The review is reported in accordance with the PRISMA 2020 statement, and this abstract follows the PRISMA 2020 for Abstracts checklist. **Results**: Eight case–control studies were included, comprising 421 IBS patients and 428 controls. Of the seven studies contributing to the primary synthesis, two reported significantly elevated zonulin in IBS, one produced opposite results with two different assays applied to the same samples, and four found no significant difference compared with controls. An eighth study, which combined IBS with inflammatory bowel disease and did not report IBS-specific data, was retained as supportive, non-primary evidence. Crucially, the studies employed different ELISA kits (ALPCO, Cusabio, Immundiagnostik, Zellbio) and different units of measurement. Independent validation studies have demonstrated that the most frequently used commercial kits do not recognise the pre-haptoglobin-2 molecule claimed to be zonulin, but instead detect haptoglobin, complement C3, or properdin. The only study employing a monoclonal antibody specific for pre-haptoglobin-2 observed lower, rather than higher, concentrations in IBS. **Conclusions**: The existing literature based on commercial ELISAs cannot determine whether pre-HP2/zonulin is altered in IBS, and the single genotype-controlled monoclonal assay suggests lower, not higher, concentrations in IBS. The disparate results are largely attributable to the application of ELISA kits that lack analytical specificity for the proposed target. Future research must use analytically validated assays, such as monoclonal antibody-based methods with concomitant genotyping for haptoglobin polymorphisms, before zonulin can be considered a meaningful biomarker in IBS. **Funding:** The Article Processing Charges were funded by the University of Medicine and Pharmacy of Craiova, Romania. **Registration:** The review was not prospectively registered; a retrospective protocol registration is available on OSF [10.17605/OSF.IO/MFA6J].

## 1. Introduction

Irritable bowel syndrome (IBS) is one of the most frequently encountered disorders in both primary care and gastroenterology clinics, characterised by chronic, recurrent abdominal pain associated with defecation or a change in bowel habits [1]. It is classified, according to the predominant stool pattern, into diarrhoea-predominant (IBS-D), constipation-predominant (IBS-C), mixed (IBS-M), and unsubtyped forms [1]. The global prevalence is estimated at approximately 4–10% depending on the diagnostic criteria applied, with a consistent female predominance and a peak incidence in young adulthood [2,3]. A recent meta-analysis of 96 studies across 52 countries reported a pooled global prevalence of 14.1% when both Rome III and Rome IV criteria were considered, with IBS-D and IBS-M being the most common subtypes [4]. The Rome Foundation Global Epidemiology Study, which surveyed 33 countries using Rome IV criteria, found that 4.1% of adults met the criteria for IBS, with higher rates among women and younger individuals [5]. Although IBS is not associated with increased mortality, it markedly impairs health-related quality of life, comparable to that seen in organic gastrointestinal diseases such as inflammatory bowel disease, and imposes a substantial socioeconomic burden through direct healthcare costs and lost productivity [6,7].

The pathophysiology of IBS is widely accepted to be multifactorial, involving a complex interplay between the gut microbiome, the enteric and central nervous systems, mucosal immune activation, and environmental factors [8,9]. The Rome IV diagnostic framework conceptualises IBS as a disorder of gut-brain interaction, in which abnormalities in bidirectional neurohumoral communication contribute to altered motility, visceral hypersensitivity, and intestinal barrier dysfunction [1,10]. The gut-brain axis comprises a dense network of neural, endocrine, and immune signalling pathways, and its dysregulation is now considered central to the development and perpetuation of IBS symptoms [11,12]. Among the various pathophysiological components, the concept of a disrupted intestinal epithelial barrier–colloquially termed “leaky gut”–has attracted substantial attention as a potential mechanistic pathway and therapeutic target [13,14].

The intestinal epithelium forms a selectively permeable interface between the external luminal environment and the internal milieu, a function mediated by the apical junctional complex composed of tight junctions, adherens junctions, and desmosomes [15]. Tight junctions, in particular, regulate the paracellular passage of ions, solutes, and larger molecules, and their integrity is influenced by a variety of endogenous and exogenous factors, including cytokines, dietary components, and microbial metabolites [16,17]. A systematic review by Hanning and colleagues identified 66 studies that evaluated barrier function in IBS and found that permeability was increased compared with healthy controls in 9 of 13 studies in IBS-D and in all four studies of post-infectious IBS, whereas only a minority of IBS-C studies demonstrated barrier dysfunction [18]. Characteristic changes in IBS include decreased expression of zonula occludens-1 (ZO-1) and occludin in colonic mucosal biopsies, as well as alterations in the distribution and phosphorylation state of claudin proteins [19,20].

The immune system plays a critical role as a master regulator of gut–brain communication, with innate and adaptive immune cells responding to luminal antigens that cross a compromised epithelial barrier [21]. In IBS, low-grade mucosal inflammation–distinct from the overt inflammation seen in inflammatory bowel disease–has been documented in a substantial subset of patients and is characterised by increased numbers of mast cells, intraepithelial lymphocytes, and elevated concentrations of pro-inflammatory cytokines including interleukin-6, interleukin-8, and tumour necrosis factor-alpha [21,22,23]. This immune activation, in turn, can sensitise enteric and afferent sensory neurons, thereby perpetuating the cycle of visceral hypersensitivity and abdominal pain that defines the syndrome [24,25]. These observations have motivated a search for reliable, non-invasive biomarkers that reflect intestinal barrier status and might aid in the diagnosis, subtyping, or therapeutic monitoring of IBS [26].

Zonulin emerged as a candidate biomarker in the early 2000s. It was initially identified as the human homologue of the Vibrio cholerae zonula occludens toxin and was proposed to be a physiological modulator of intercellular tight junctions that reversibly increases intestinal permeability [27]. The mature zonulin protein was subsequently shown to correspond to pre-haptoglobin-2 (pre-HP2), the inactive precursor of haptoglobin-2, a polymorphic acute-phase protein involved in scavenging free haemoglobin [28]. Zonulin binds to the epidermal growth factor receptor and protease-activated receptor 2 on the apical surface of enterocytes, initiating a cascade of intracellular signalling that results in the transient phosphorylation and disassembly of tight junction proteins, thereby increasing paracellular permeability [29]. Because expression of pre-HP2 depends on the presence of the Haptoglobin 2 (HP2) allele at the haptoglobin gene locus, individuals homozygous for the Haptoglobin 1 (HP1) allele (genotype HP1-1) are genetically incapable of producing the protein [30]. This genetic restriction has become a critical consideration in evaluating the validity of commercial zonulin assays.

Zonulin has been reported to be elevated in several conditions characterised by tight junction disruption, most notably celiac disease, where gliadin-induced zonulin release from enterocytes leads to a cascade of barrier loss and adaptive immune activation [31,32]. Gliadin binds to the C-X-C chemokine receptor type 3 (CXCR3) on intestinal epithelial cells, triggering a Myeloid differentiation primary response 88 (MyD88)-dependent signalling pathway that culminates in zonulin release [33]. The therapeutic relevance of this pathway has been demonstrated in clinical trials of larazotide acetate, a synthetic peptide that antagonises zonulin and has been shown to prevent tight junction opening and ameliorate symptoms in non-responsive celiac disease [34,35]. These observations, together with reports of elevated zonulin in type 1 diabetes, obesity, and other metabolic diseases, raised the compelling hypothesis that circulating zonulin might also serve as a non-invasive indicator of gut permeability in IBS, a condition in which serial intestinal biopsy is clinically impractical [36,37].

Several enzyme-linked immunosorbent assay (ELISA) kits targeting zonulin have been commercialised and have been widely used in IBS research. However, beginning in 2018, a series of stringent analytical studies demonstrated that the most widely used kits do not in fact detect zonulin or pre-HP2, but instead recognise other serum proteins including haptoglobin, complement component C3, and properdin [38,39]. A subsequent letter by Massier and colleagues reinforced these concerns, arguing that the commercial zonulin ELISA is neither adequate to measure intestinal permeability nor the postulated biomarker zonulin, and that the continued use of these kits has blurred the picture in leaky gut research [40]. These findings have cast a shadow over the entire extant literature and have made a qualitative re-evaluation of the clinical studies an urgent necessity.

The present systematic review was conducted to answer the focused question: “Are zonulin levels increased in IBS patients compared with healthy controls?” In recognition of the marked analytical heterogeneity among the eligible studies and, more importantly, the fundamental uncertainty surrounding the identity of the analyte being measured by the commercial kits, a quantitative meta-analysis was deemed inappropriate. A narrative synthesis of the evidence is instead provided, with particular emphasis on how assay methodology has shaped the published findings.

## 2. Materials and Methods

### 2.1. Search Strategy and Study Selection

A systematic search of PubMed was performed from database inception to April 2026 using the following comprehensive search strategy: (“zonulin”[tiab] OR “pre-haptoglobin-2”[tiab] OR “prehaptoglobin 2”[tiab] OR “HP2”[tiab] OR “haptoglobin 2”[tiab]) AND (“Irritable Bowel Syndrome”[mh] OR “irritable bowel syndrome”[tiab] OR “IBS”[tiab]) NOT (“animals”[mh] NOT “humans”[mh]). The most recent study meeting the eligibility criteria was published in June 2024. This strategy was designed to capture records that used any of the common nomenclatures for the zonulin protein in conjunction with terms for IBS.

Studies were included if they met the following pre-specified criteria: (a) enrolled adult patients (≥18 years) diagnosed with IBS according to standardised symptom-based criteria, specifically Rome III or Rome IV; (b) included a comparison group of healthy controls explicitly defined as free from gastrointestinal or significant systemic inflammatory diseases; (c) measured zonulin concentrations in serum or plasma using an ELISA method; and (d) reported quantitative data suitable for comparison between groups. Exclusion criteria were: case reports, narrative reviews, animal studies, conference abstracts without full-text companion papers, studies lacking a healthy control group, and studies in which the population included mixed functional gastrointestinal disorders without extractable IBS-specific data.

**Figure 1:**
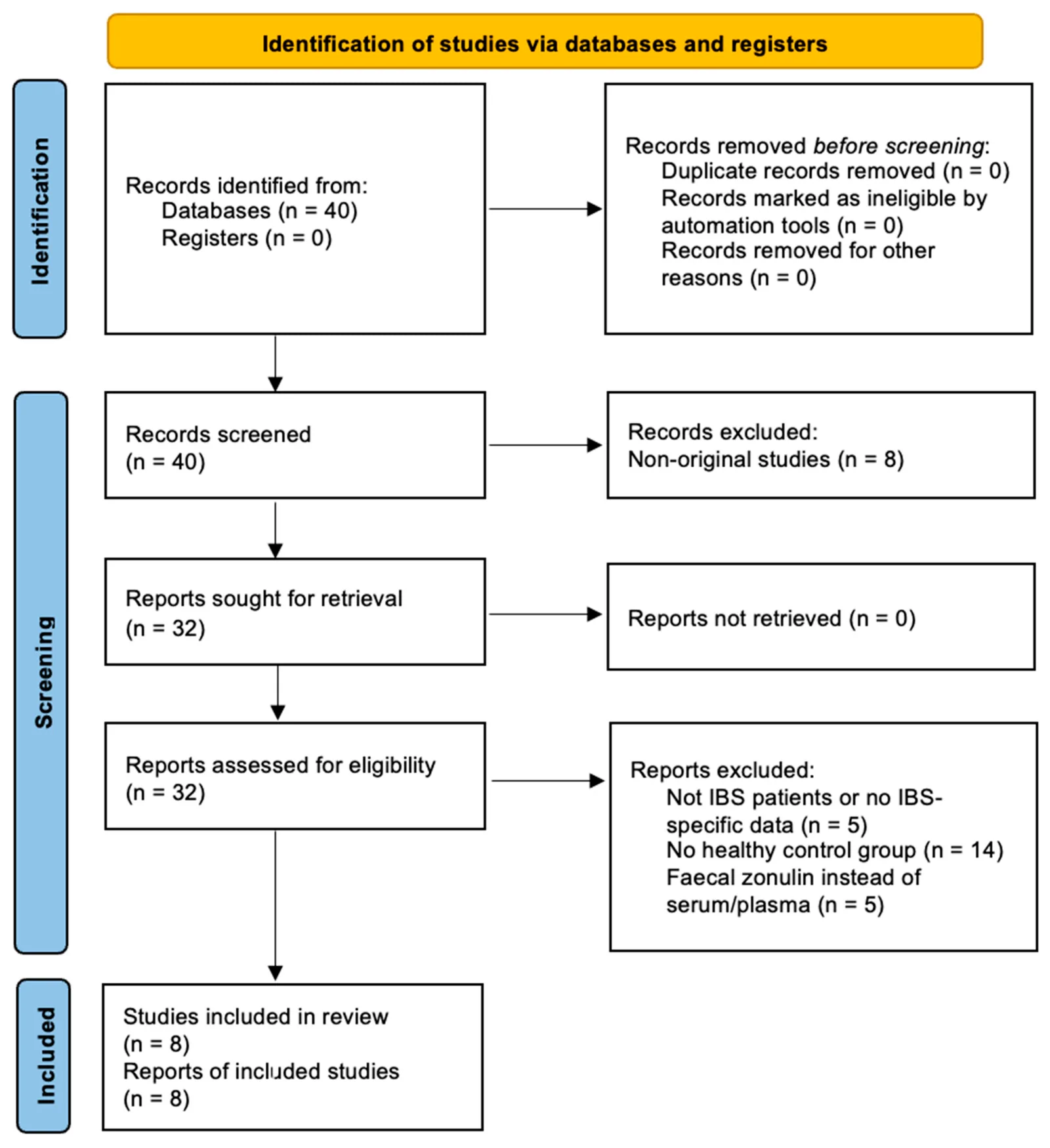
Flow diagram of study selection process.

The initial literature search yielded 40 records, 9 articles being excluded since they were non-original studies (reviews, commentaries, protocols), with a remaining number of 31 full-text articles to be assessed for eligibility. Of these, 23 were excluded: 5 because they did not include IBS patients or IBS-specific data, 13 because they did not include a healthy control group, and 5 that studied faecal zonulin instead of serum/plasma. Ultimately, eight full-text articles fulfilled all inclusion criteria and were retained for the systematic review [41,42,43,44,45,46,47,48] as presented in Figure 1.

Records were screened by one reviewer (I.C.M.), and a second reviewer (C.M.M.) verified the excluded records and full-text assessments. Disagreements were resolved by discussion. No automation tools were used in the screening process. The selection process is summarised in Figure 1.

### 2.2. Data Extraction and Synthesis

Data were extracted by one reviewer (I.C.M.) using a standardised data collection form, and a second reviewer (C.M.M.) verified the extracted data; discrepancies were resolved by discussion. No study investigators were contacted, missing or unclear data were recorded as not reported, and no automation tools were used. The following information was extracted from each study: first author and year, country, study design, number of IBS and control participants, IBS subtype distribution, diagnostic criteria, ELISA kit manufacturer and catalogue information, biological matrix (serum or plasma), reporting units (absolute ng/mL, normalised pg/mg total protein, or arbitrary units), mean or median zonulin concentrations with measures of dispersion, direction and statistical significance of between-group comparisons, and any reported correlations with clinical symptom scores. Dietary intake was not among the extracted variables because only one of the eight included studies reported it, and none reported a controlled analysis of diet as a confounder.

The primary outcome was the between-group difference in serum or plasma zonulin concentrations between adults with IBS and healthy controls. Secondary outcomes were correlations between zonulin concentrations and clinical parameters, and differences according to IBS subtype, assay kit, biological matrix, or reporting units. Because no meta-analysis was performed, effect measures are presented descriptively as differences in means or medians (with standard deviations or interquartile ranges, as reported) accompanied by *p*-values, and as correlation coefficients (r) for symptom associations. No pooled effect measure was calculated. Any pooled estimate would have amalgamated signals from different molecular targets with varying degrees of non-specificity, yielding a result uninterpretable from a biological perspective.

Studies were eligible for the primary synthesis if they reported a between-group comparison of serum or plasma zonulin between IBS patients and healthy controls; mixed IBS/Inflammatory bowel disease (IBD) studies without IBS-specific data were excluded from the primary synthesis. Results are tabulated in Table 1 (study characteristics), Table 2 (assay validation), and Table 3 (risk of bias), and the selection process is shown in Figure 1; no forest plots were generated. Heterogeneity was explored narratively by grouping studies according to ELISA kit, sample matrix, reporting units, IBS subtype, and availability of independent assay validation.

No data conversions or imputations were performed; concentrations are presented in their original units, and where a study did not report a measure of dispersion or IBS-specific summary statistics, this was recorded as not reported without assumptions about missing data. Given the absence of a pooled estimate and the small number of included studies, no sensitivity analyses were performed; the robustness of the conclusions was instead examined narratively by separating studies according to the availability of independent analytical validation for the assay used. No formal assessment of reporting bias was performed, although all included studies reported the primary outcome and no evidence of selective outcome reporting was identified. No formal certainty assessment using Grading of Recommendations Assessment, Development and Evaluation (GRADE) was performed. GRADE’s domains assume that the outcome was measured with a valid instrument, which the central finding of this review contradicts; the commercial ELISA kits do not measure the intended target. Certainty was therefore assessed narratively and rated very low, primarily because all included studies used these non-specific kits and because the studies were methodologically heterogeneous.

### 2.3. Risk of Bias Assessment

The methodological quality and risk of bias of the included case–control studies were assessed using the Newcastle–Ottawa Scale (NOS) for case–control studies. The NOS evaluates three domains: selection of study groups (maximum 4 points), comparability of groups (maximum 2 points), and ascertainment of exposure (maximum 3 points), yielding a maximum score of 9. A score of 7–9 was considered low risk of bias, 4–6 intermediate risk, and less than 4 high risk. Two reviewers (I.C.M. and C.M.M.) independently assessed each study, with disagreements resolved by consensus. The detailed scoring for each study is presented in Table 3.

### 2.4. Registration and Protocol

This review was not prospectively registered. The review protocol was retrospectively registered on the Open Science Framework (OSF) Registries [10.17605/OSF.IO/MFA6J] after completion of the review, in order to provide a permanent, timestamped record of the eligibility criteria, search strategy, data extraction items, risk-of-bias method, and synthesis approach reported in this manuscript. OSF was selected because PROSPERO does not accept reviews for which data extraction has already been completed, and OSF accepts retrospective registration. No protocol was prepared prior to the conduct of the review, and no amendments to a prior protocol are therefore applicable.

## 3. Results

### 3.1. Characteristics of Included Studies

Of the 40 records screened, 8 were excluded as non-original studies (reviews, commentaries, protocols), and of the 32 full-text articles assessed for eligibility, 24 were excluded: 5 had no IBS-specific data, 14 lacked a healthy control group, and 5 measured faecal rather than serum/plasma zonulin. A complete list of all excluded studies, with reasons and DOIs, is provided in Supplementary Table S1.

The eight eligible case–control studies, comprising 421 IBS patients and 428 controls, were published between 2018 and 2022, a period that coincides with the growing interest in non-invasive biomarkers of intestinal permeability within the functional gastrointestinal disorders field. The studies originated from geographically diverse settings: Iran [41], the United States [42], Italy [44,46], Russia [45], Sweden [47], Bulgaria [48], and one multicentre effort involving both Australia and Sweden [43]. One of the eight included studies (Kushlinskii et al. [45]) was retained as supportive, non-primary evidence because it did not report IBS-specific summary statistics and did not specify the diagnostic criteria applied for IBS. This study is marked accordingly in Table 1 and is described separately in Section 3.2. A summary of their characteristics is presented in Table 1.

The sample sizes of the IBS groups varied widely, from as few as 15 patients in the study by Peruhova et al. [48] to 78 patients in the study by Meira-de-Faria et al. [47]. Most studies focused on patients with IBS-D, consistent with the generally stronger evidence for permeability alterations in this subgroup; however, Singh et al. [42] also included a sizeable IBS-C cohort, and Talley et al. [43] and Meira-de-Faria et al. [47] combined multiple IBS subtypes. All studies relied on standardised Rome criteria for case definition and explicitly excluded organic gastrointestinal diseases through a combination of serological testing, endoscopy, and histology where appropriate.

Four distinct commercial ELISA kits were utilised across the eight studies: Cusabio (three studies [44,47,48]), Immundiagnostik (two studies [45,46]), ALPCO (one study [42]), and Zellbio (one study [41]). The kit used by Talley et al. [43] was not reported in the original publication and is recorded as not reported in Table 1. Reporting units differed substantially. Some studies expressed zonulin as an absolute concentration in ng/mL [41,42,45,46,47,48], while others normalised concentrations to total serum protein and reported values in pg/mg [43,44]. This inter-study variation in data presentation, even before the question of analytical validity is considered, precluded any meaningful quantitative pooling. Because serum and plasma are not analytically interchangeable and because ng/mL and pg/mg total protein are not directly convertible, the absolute zonulin concentrations reported in these studies should not be pooled or directly compared across studies; they are presented in their original units and matrices throughout this review.

### 3.2. Zonulin Levels in IBS Versus Controls

No statistical synthesis (meta-analysis) was performed; therefore, no pooled summary estimate, confidence interval, or measure of statistical heterogeneity is presented. The findings below are described narratively, with the direction of effect indicated for each study.

The primary synthesis of seven studies was divided: two studies reported significantly elevated zonulin in IBS using commercial ELISA kits (Singh et al. [42], Peruhova et al. [48]), one study produced opposite results with two different assays (Meira-de-Faria et al. [47]), and four studies found no significant difference (Rezazadegan et al. [41], Talley et al. [43], Barbaro et al. [44], Linsalata et al. [46]).

The eight contributing studies were all case–control in design and were published between 2018 and 2022. Six were judged to be at low risk of bias (NOS 7–9), and two at intermediate risk (NOS 4–6); no study was at high risk. The three studies reporting elevated zonulin used ALPCO, Cusabio, or Immundiagnostik kits, whereas the four null studies used Zellbio, Cusabio, or Immundiagnostik kits. Reporting units differed (ng/mL vs. pg/mg total protein), and IBS subtype distribution was inconsistent across studies. Two studies concluded that zonulin levels were significantly elevated in IBS (Singh et al. [42] and Peruhova et al. [48]; n = 115 IBS patients and 50 controls), while one study (Meira-de-Faria et al. [47]) produced opposite results with two different assays applied to the same samples and is presented as a dual-result study. Singh et al. [42] reported one of the most comprehensive analyses in this group. Using the ALPCO ELISA, they found that median serum zonulin in IBS-D patients (65.8 ng/mL, interquartile range [IQR] 43.3–93.4) and in IBS-C patients (67.2 ng/mL, IQR 44.2–91.2) was significantly higher than in healthy controls (45.2 ng/mL, IQR 27.3–61.5; *p* = 0.009 and *p* = 0.006, respectively). Notably, the zonulin concentrations in IBS were statistically indistinguishable from those observed in patients with active celiac disease (66.7 ng/mL, IQR 55.2–80.5), a disease in which tight junction dysfunction is well established. The authors further demonstrated that, within the IBS-D subgroup, zonulin levels correlated positively with stool frequency per week (r = 0.45; *p* = 0.03) and with the severity of dissatisfaction with bowel habits (r = 0.43; *p* = 0.007) on the IBS Severity Scoring System (IBS-SSS), suggesting a potential clinical relevance.

Meira-de-Faria et al. [47] reported that plasma zonulin, as measured by the Cusabio kit, was significantly higher in 78 IBS patients than in 40 healthy controls (*p* < 0.001). Similarly, the smaller Bulgarian study by Peruhova et al. [48] found elevated mean serum zonulin in 15 IBS patients compared with eight controls using the same Cusabio kit (0.378 ± 0.13 vs. 0.250 ± 0.14 ng/mL; *p* = 0.0315). Because the two assays applied to the same samples produced opposite results, this study cannot be classified as either positive or negative; it is presented as a dual-result study and is the most instructive single piece of evidence in the review.

In contrast, four studies (n = 199 IBS patients and 288 controls) found no significant difference between IBS and control populations. Rezazadegan et al. [41], in a well-matched case–control study from Iran using the Zellbio kit, reported that mean serum zonulin was 15.06 ± 3.86 ng/mL in 61 newly diagnosed IBS-D patients and 14.96 ± 3.71 ng/mL in 61 matched healthy controls (*p* = 0.888). This study was methodologically notable for its rigorous exclusion of confounders, including dietary supplement use and recent antibiotic exposure, and for its concurrent assessment of zinc nutritional status, which was the primary focus of the investigation.

Talley et al. [43] published the largest study in this field, drawing on both a population-based cohort of 242 individuals and a hospital-based cohort of 102 individuals from Australia and Sweden. Serum zonulin was expressed in pg/mg total protein. In the population-based sample, mean zonulin was 3999.8 pg/mg in IBS patients and 3755.1 pg/mg in healthy controls (*p* = 0.5). In the clinic-based sample, mean zonulin was 3577.9 pg/mg in IBS patients and 3407.2 pg/mg in patients with organic gastrointestinal diseases (*p* = 0.7), indicating that zonulin failed to discriminate IBS from either health or other gastrointestinal pathology. The study also found no elevation of zonulin in functional dyspepsia or non-coeliac wheat sensitivity. In conclusion, only the population-based cohort contributed to the primary IBS-versus-healthy-control synthesis (40 IBS patients, 182 healthy controls). The clinic-based cohort of the same study compared IBS patients with patients with organic gastrointestinal diseases rather than with healthy controls, and is therefore reported as a secondary observation only.

Barbaro et al. [44] conducted a multicentre Italian study focused primarily on non-coeliac gluten sensitivity (NCGS), in which IBS-D served as a comparator group. Serum zonulin, measured by the Cusabio kit and normalised to total protein, was 7.9 ± 10.7 pg/mg in 59 IBS-D patients and 5.5 ± 5.5 pg/mg in 25 asymptomatic controls, a difference that did not approach statistical significance. By contrast, zonulin was markedly and significantly elevated in both NCGS (35.8 ± 73.9 pg/mg) and celiac disease (33.3 ± 16.2 pg/mg) compared with both IBS-D and controls, suggesting that, within the analytical framework of this particular kit, IBS was not associated with a detectable zonulin signal. Linsalata et al. [46] similarly found no difference in zonulin between 39 IBS-D patients and 20 healthy controls when using the Immundiagnostik assay, although zonulin was significantly elevated in celiac disease patients.

Kushlinskii et al. [45], employing the Immundiagnostik assay, observed that the pooled pathological control group, which included 29 IBS patients together with patients with ulcerative colitis and Crohn’s disease, had significantly higher zonulin than healthy controls (*p* < 0.0002). This study could not contribute an IBS-specific estimate to the primary synthesis because IBS-specific summary statistics were not reported and the diagnostic criteria used for IBS were not specified. It is therefore presented as supportive evidence only, and the selection-bias implications of its mixed cohort are discussed in Section 3.4.

Correlations between zonulin concentrations and clinical parameters were examined in several studies, with inconsistent results (summarised in Supplementary Table S2). In IBS-D patients, Singh et al. [42] reported a positive correlation between serum zonulin and stool frequency per week (r = 0.45; *p* = 0.03) and between zonulin and dissatisfaction with bowel habits (r = 0.43; *p* = 0.007), but no correlation with the overall IBS-SSS score (r = 0.24; *p* = 0.15). Rezazadegan et al. [41] found no correlation between serum zonulin and the IBS-SSS total score in their IBS-D cohort (r = −0.061; *p* = 0.640). Peruhova et al. [48] reported no significant relationship between zonulin concentrations and gastrointestinal symptom scores, although no correlation coefficients were provided. Barbaro et al. [44] found no significant relationship between zonulin and clinical data in their IBS-D cohort, although correlations were observed in the non-coeliac gluten sensitivity and coeliac disease groups. Linsalata et al. [46] reported no correlation between gastrointestinal symptom scores and any serum biomarker. Meira-de-Faria et al. [47] stated that no correlation with IBS-specific symptoms was found, but did not report the values. Talley et al. [43] and Kushlinskii et al. [45] did not report correlation analyses. Across studies, only Singh et al. detected statistically significant correlations, and these were confined to the IBS-D subgroup and to stool-related parameters rather than to global symptom severity. The small number of patients with severe symptoms in several cohorts may have limited statistical power to detect associations.

### 3.3. Validity of Commercial Zonulin ELISA Kits

A critical finding of this review is that the contradictory results between studies are best understood when the analytical specificity of the ELISA kits used is scrutinised. Two independent validation studies contributed to this synthesis. Both were laboratory-based analytical studies rather than clinical case–control studies, and formal NOS assessment was therefore not applicable. Their methodological quality was high, employing mass spectrometry, immunoprecipitation, recombinant protein testing, and haptoglobin genotyping. The first indications that the commercial kits might not be measuring zonulin came from a 2018 paper by Scheffler and colleagues, in which they observed that zonulin concentrations did not correlate with haptoglobin genotypes in a large German cohort, publishing a landmark investigation of the Immundiagnostik zonulin ELISA [38]. Using a combination of Western blotting, mass spectrometry, and immunoprecipitation, they demonstrated that the polyclonal antibody at the core of the kit did not bind to pre-haptoglobin-2 or any haptoglobin-related protein. Instead, the antibody recognised properdin, a positive regulator of the alternative complement pathway. The authors noted that properdin is an acute-phase reactant, and its circulating concentrations are influenced by inflammatory conditions independent of intestinal permeability.

One year later, Ajamian et al. [39] extended this analysis to include the Cusabio kit, the most frequently used assay in the IBS literature (Table 2). Their study, which employed recombinant zonulin, haptoglobin phenotyping by starch gel electrophoresis, and commercial complement and haptoglobin ELISAs, reached similarly concerning conclusions. Neither the Immundiagnostik nor the Cusabio kit was able to detect recombinant zonulin. The Immundiagnostik kit correlated most strongly with haptoglobin concentration and complement C3, while the signal from the Cusabio kit was predominantly attributable to complement C3 with a smaller contribution from haptoglobin. Crucially, in individuals who were haptoglobin 1-1 homozygotes–who should have no circulating pre-HP2 or zonulin by genetic definition–both kits returned readily detectable signals, confirming that the measured analyte was not zonulin. Massier and colleagues subsequently underscored these findings, arguing in a commentary in Gut that using the commercial zonulin ELISA is neither adequate to measure intestinal permeability nor the postulated biomarker zonulin, and that the continued use of these kits has blurred the picture in leaky gut research [40].

The Meira-de-Faria study [47] provided a powerful within-study illustration of the consequences of using these kits. The authors measured zonulin in the same cohort of 78 IBS patients and 40 healthy controls using both the Cusabio ELISA and a monoclonal antibody-based assay specific for pre-HP2 (kindly provided by Bio-Rad). The Cusabio kit indicated that IBS patients had significantly higher zonulin levels than controls. However, when the same samples were assayed with the pre-HP2-specific monoclonal antibody, the results were diametrically opposite: pre-HP2 concentrations were significantly lower in IBS patients than in healthy controls. Moreover, the monoclonal antibody-based ELISA produced zero signal in all individuals genotyped as HP1-1, demonstrating perfect concordance with the known genetics of pre-HP2 expression. The Cusabio kit, by contrast, showed no respect for haptoglobin genotype, with HP1-1 individuals displaying zonulin levels well within the range of HP2 carriers.

The ALPCO kit, used in the positive study by Singh et al. [42], has not been subjected to the same detailed analytical characterisation as the Immundiagnostik and Cusabio kits. However, it is also a polyclonal antibody-based competitive ELISA and was developed using the same immunising peptide sequence (GGVLVQPG) that forms the basis of the Immundiagnostik kit [38]. This sequence is absent from the pre-HP2 molecule and is more consistent with an immunoglobulin heavy-chain variable region [28,38]. Although definitive proof of non-specificity for the ALPCO kit is lacking, the common lineage of the commercial kits raises a strong presumption that similar cross-reactivity issues apply.

Taken together, these validation studies demonstrate that the four studies that reported elevated zonulin in IBS all employed kits that either have been proven to detect proteins other than zonulin (Cusabio, Immundiagnostik) or have not undergone rigorous independent target verification (ALPCO). The four studies that found no difference also used these kits or the Zellbio kit, about which even less is known. Therefore, these observations indicate that seven of the eight included studies cannot reliably inform us about circulating zonulin concentrations in IBS, because the commercial kits they used do not measure the intended analyte. The remaining study, Meira-de-Faria et al. [47], used a validated monoclonal antibody specific for pre-HP2 and provides preliminary evidence that pre-HP2 concentrations are lower, rather than higher, in IBS. This finding is based on a single cohort of 78 IBS patients and 40 controls, did not correlate with colonic permeability, and requires replication before firm conclusions can be drawn.

Importantly, the ALPCO and Zellbio kits, both of which were used in included clinical studies, have not been subjected to the same detailed independent analytical characterisation as the Immundiagnostik and Cusabio kits. The ALPCO kit is a polyclonal antibody-based competitive ELISA developed using the same GGVLVQPG immunising sequence as the Immundiagnostik kit [38], which raises a strong presumption of similar non-specificity, although definitive proof is lacking. For the Zellbio kit, even less is known, and no independent analytical validation has been published.

### 3.4. Risk of Bias Assessment

The risk of bias assessment of the eight included case–control studies is summarised in Table 3. Six studies were judged to be at low risk of bias (NOS score 7–9): Rezazadegan et al. [41] (8), Singh et al. [42] (7), Talley et al. [43] (9), Barbaro et al. [44] (7), Linsalata et al. [46] (7), and Meira-de-Faria et al. [47] (8). Two studies were judged to be at intermediate risk of bias (NOS score 4–6): Kushlinskii et al. [45] (4) and Peruhova et al. [48] (5). No study was judged to be at high risk of bias. In addition to the NOS assessment, a customised assay-specific risk-of-bias domain was applied to all eight included studies (Table S3). Only one study (Meira-de-Faria et al. [47]) was judged to be at low concern across all assay-specific items, because it used a validated monoclonal antibody specific for pre-HP2 and performed haptoglobin genotyping on all participants. The remaining seven studies were judged to be of great concern, primarily because the kits they used have been shown to lack analytical specificity for the intended target. Only one study reported blinding of laboratory personnel [44], and no study reported batch effects. Haptoglobin genotyping was performed in only one study [47]. These findings reinforce the conclusion that the dominant threat to validity in this literature is analytical rather than conventional methodological bias.

The most consistent limitation across studies was the failure to report the non-response rate. In the comparability domain, Kushlinskii et al. [45] and Peruhova et al. [48] did not describe adjustment for potentially important confounders. Selection bias was a particular concern in the study by Kushlinskii et al. [45], which was retained as supportive, non-primary evidence and combined IBS patients with inflammatory bowel disease patients in a single pathological group, and in Peruhova et al. [48], which enrolled a small sample and did not report IBS subtype distribution. Importantly, none of the studies was judged to be at high risk of bias, and the overall quality of the included evidence was therefore moderate to high. However, the dominant threat to the validity of the entire body of evidence is not conventional methodological bias but rather the analytical non-specificity of the ELISA kits used.

No formal assessment of reporting bias was performed because no meta-analysis was conducted and the number of included studies was small. No formal GRADE assessment was performed, for the reasons stated in Section 2.2. Certainty was rated narratively as very low: conventional risk of bias was moderate to high (six studies low, two intermediate on the NOS), but assay-specific concern was great in seven of eight studies (Table S3), and the outcome itself was not validly measured because the assays lack specificity for the intended target.

## 4. Discussion

This systematic review of eight case–control studies reveals a literature that appears divided on the question of whether zonulin is elevated in IBS, but this division does not reflect genuine clinical or biological heterogeneity. Rather, it is more plausibly explained as an artefact of the analytical methods deployed.

The commercial ELISA kits that have been the exclusive tools of zonulin measurement in the IBS literature do not measure zonulin. They measure a variable mixture of haptoglobin, complement factor C3, and properdin–all of which are acute-phase proteins whose serum concentrations are influenced by a wide range of inflammatory, metabolic, and infectious processes that may or may not be present in any given cohort of IBS patients and controls.

The molecular basis for this mis-targeting lies in the history of zonulin research. In the year 2000, Wang and colleagues [27] reported the partial purification of a protein from human intestinal tissue that exhibited tight-junction-modulating activity. Amino-terminal sequencing of this protein yielded the octapeptide GGVLVQPG. Antibodies raised against this peptide were used to develop the first commercial assays. Eight years later, Tripathi and colleagues [28] identified the zonulin protein as pre-HP2 through proteomic analysis. Critically, the GGVLVQPG sequence is not present in pre-HP2. The original peptide sequence instead aligns most closely with the variable region of immunoglobulin heavy chains, suggesting that the protein originally isolated by Wang et al. may have been an immunoglobulin fragment co-purifying with the true tight-junction-regulating factor [38]. Despite this sequence discrepancy being published in 2009, the commercial kits continued to employ the original GGVLVQPG peptide as the immunogen, with the result that the antibodies they contain are directed against a structurally unrelated set of serum proteins.

Further complicating the picture is the biological complexity of the haptoglobin–zonulin system itself. Haptoglobin is a haemoglobin-binding protein with immunomodulatory properties, and its gene harbours a common polymorphism with two principal alleles, HP1 and HP2, giving rise to three principal phenotypes (HP1-1, HP2-1, HP2-2) with distinct functional properties [30,49]. The HP2-2 phenotype has been associated with an unfavourable disease course in several inflammatory and autoimmune disorders [50]. Circulating haptoglobin concentrations are not only genetically determined but also fluctuate as part of the acute-phase response, rising precipitously during infection, injury, and systemic inflammation [49]. Complement component C3 and properdin are similarly acute-phase reactants and participate in a host of inflammatory cascades entirely unrelated to intestinal tight junction regulation. Any assay that inadvertently captures these proteins will therefore generate a signal that is profoundly confounded by intercurrent infection, metabolic status, psychological stress, smoking, and a range of subclinical inflammatory states, rendering it unsuitable as a specific index of gut permeability.

The continued use of these kits despite published concerns likely reflects their commercial availability, low cost, technical simplicity, and manufacturer claims of specificity that are rarely independently verified, together with the absence of a widely available validated alternative. To reinforce the central argument of the present review, the continued use of these kits risks perpetuating findings that reflect cross-reactive acute-phase proteins rather than zonulin. The clinical implications of measuring acute-phase reactants instead of a specific tight-junction modulator are substantial. Serum haptoglobin, complement C3, and properdin all rise during systemic inflammation, infection, surgical stress, and in response to a variety of chronic diseases. Even subclinical inflammatory states–such as those associated with obesity, metabolic syndrome, psychological stress, or smoking–can shift the concentrations of these proteins. IBS, by its current definition, is a non-inflammatory disorder, but studies have repeatedly shown that a subset of patients exhibit low-grade mucosal inflammation, particularly those with post-infectious IBS [9,23]. It is entirely plausible that the “zonulin” signal reported in some IBS studies reflects not a specific tight-junction defect, but a subtle, non-specific elevation in acute-phase reactants related to the pro-inflammatory milieu that characterises this subgroup. This would explain why studies observing an elevation did so inconsistently across kits and why correlations with gastrointestinal symptoms were sporadic at best.

The study by Meira-de-Faria et al. [47] is particularly instructive. It demonstrates, within a single patient and control cohort, that the choice of assay can completely invert the study conclusion. The Cusabio kit reported a significant increase in IBS; the monoclonal pre-HP2 antibody reported a significant decrease. The latter finding, if replicated, would itself be of considerable biological interest, potentially suggesting that pre-HP2 is consumed or down-regulated in the context of chronic barrier dysfunction, or that IBS is associated with a shift in haptoglobin genotype distribution. However, because this is the only study to have employed a validated monoclonal assay, it would be premature to draw firm conclusions. What can be stated with confidence is that the monoclonal antibody-based approach, which showed complete concordance with haptoglobin genotyping (zero values in HP1-1 individuals), represents the analytical gold standard against which all future work should be judged [47,51].

The limitations of the primary studies included in this review extend beyond the assay problem and must therefore be acknowledged. Our conclusions rest on only eight eligible case–control studies, comprising 421 IBS patients and 428 controls, with considerable variation between studies in sample size, IBS subtype distribution, assay kit, reporting units, and biological matrix. Given this small evidence base and the analytical non-specificity of the assays used, the conclusions should be interpreted with caution and may not be generalisable to all IBS populations or clinical settings. Sample sizes were generally modest: only two studies enrolled more than 60 IBS patients [41,47], and one enrolled only 15 [48]. Most cohorts were drawn from tertiary referral centres, which may over-represent patients with more severe or complex phenotypes. The classification of IBS subtypes was inconsistent, with some studies focusing exclusively on IBS-D, others including IBS-C and IBS-M, and some not reporting subtype distribution at all. The heterogeneity of reporting units–ng/mL in some studies, pg/mg total protein in others–and of biological matrices–serum in seven studies and plasma in one–precluded any quantitative comparison of absolute concentrations across studies. Serum and plasma are not analytically interchangeable, and the effect of the clotting process on the detectable analyte is unknown for the non-specific kits used. Absolute concentrations should therefore not be pooled or directly compared across studies, and the values are presented in their original units and matrices throughout this review. Furthermore, no study systematically controlled for known confounders of acute-phase reactant levels, such as body mass index, smoking status, psychological comorbidity, or recent infection–all of which are prevalent in the IBS population [6,43,44]. The search was restricted to a single database (PubMed). Embase, Scopus, Web of Science, Cochrane Library, and trial registries were not searched. Although PubMed indexes the core biomedical literature relevant to this review, a single-database search may have missed eligible studies indexed elsewhere. This is acknowledged as a limitation. One included study (Kushlinskii et al. [45]) was retained as supportive evidence despite not reporting IBS-specific summary statistics and not specifying the diagnostic criteria used for IBS. Retaining this study preserved transparency and enabled assessment of the Immundiagnostik kit, but it could not contribute to the primary synthesis. This reflects a pragmatic trade-off between strict adherence to the eligibility criteria and the value of transparently reporting a study identified by the search. Gender was not analysed as a subgroup variable or confounder, and no included study reported gender-stratified zonulin concentrations in IBS patients; only one study reported a sex difference in zonulin among healthy controls [1]. The review was not prospectively registered, and the retrospective OSF registration does not provide the bias-reduction benefits of prospective registration; readers should weigh the conclusions accordingly. Dietary intake was assessed in only one of the eight included studies (Rezazadegan et al. [2], which examined dietary zinc intake and found no significant correlation with serum zonulin), and no study controlled for diet as a confounder in the IBS versus control comparison. Given the known influence of diet on intestinal permeability and on circulating acute-phase protein concentrations, this represents an additional uncontrolled confounder that future studies should address. Screening and data extraction were not performed in full duplicate by two independent reviewers. One reviewer screened all records and extracted all data, and a second reviewer verified the excluded records, the full-text assessments, and the extracted data. Although this verification step provided a secondary check, full independent duplicate screening and extraction are the preferred standard for systematic reviews. This is acknowledged as a limitation.

Conventional risk-of-bias tools such as the Newcastle–Ottawa Scale do not capture analytical specificity, sample handling, batch effects, blinding, or genotyping, all of which are directly relevant to the interpretation of a biomarker review. We therefore supplemented the NOS with a customised assay-specific risk-of-bias domain (Table S3), which identified seven of eight studies as being at great concern for assay-related validity.

The implications for the field are clear and urgent. The weight of analytical evidence indicates that the published IBS–zonulin literature is largely built on measurements of the wrong proteins, with one exception: the single study that used a validated monoclonal antibody specific for pre-HP2 [47]. A reappraisal of this literature is necessary, and the inclusion of such studies in future meta-analyses or clinical guidelines should be approached with extreme caution, if not avoided altogether. For the field to advance, future investigations must adopt the following methodological standards. First, the assay used must have its target identity verified by independent means, preferably through monoclonal antibodies specific for pre-HP2 such as those described by Flanagan et al. [51] and employed by Meira-de-Faria et al. [47]. Second, all study participants must be genotyped for the HP1 and HP2 alleles, and the assay must produce zero signal in HP1-1 individuals. Third, investigators should report zonulin concentrations both as absolute values and normalised to total protein to facilitate comparison across studies. Fourth, objective measures of intestinal permeability, such as the multi-sugar urinary recovery test or Ussing chamber analysis of mucosal biopsies, should be performed concurrently to establish whether any detected pre-HP2 signal correlates with functional barrier status [52]. Fifth, future studies should consider evaluating a panel of barrier function biomarkers–including intestinal fatty acid-binding protein (I-FABP), diamine oxidase, citrulline, and lipopolysaccharide-binding protein–rather than relying on any single marker in isolation [26,53].

Faecal zonulin has been proposed as a potentially more direct reflection of intestinal luminal events, and some studies have reported associations with dietary factors, psychological parameters, and symptom severity, whereas others have found no differences [54,55,56,57]. However, the same commercial kits that lack analytical specificity for zonulin are frequently applied to faecal matrices without independent validation, and the findings are as inconsistent as those for serum. Until assays with verified target specificity are applied to faecal samples, faecal zonulin should be regarded as an exploratory research tool rather than a validated biomarker of intestinal permeability in IBS.

## 5. Conclusions

The existing IBS–zonulin literature based on commercial ELISAs cannot answer whether pre-HP2/zonulin is altered in IBS, because the kits used in these studies have not been shown to measure the intended analyte and instead detect haptoglobin, complement component C3, or properdin. Of the seven studies contributing to the primary synthesis, two reported elevated concentrations using commercial kits, one produced opposite results with a commercial kit and a validated monoclonal antibody applied to the same samples, and four found no significant difference; an eighth study could not contribute IBS-specific data and was retained as supportive evidence only. The only genotype-controlled monoclonal assay used in this literature suggests lower, not higher, pre-HP2 concentrations in IBS, in direct opposition to the commercial kit result from the same samples. Because this finding is based on a single cohort and did not correlate with colonic permeability, it requires replication before firm conclusions can be drawn. Future research must abandon the non-specific commercial kits and instead employ analytically validated assays, ideally monoclonal antibody-based methods with concomitant haptoglobin genotyping, and should measure pre-HP2/zonulin directly alongside objective measures of intestinal permeability. Only then will it be possible to determine whether the zonulin/pre-HP2 pathway genuinely contributes to the pathogenesis of IBS.

## Figures and Tables

**Table 1:** Characteristics of studies comparing serum or plasma zonulin concentrations between adults with irritable bowel syndrome (IBS) and healthy controls (HC).

Author, Year	Country	Study Design	IBS Subtype (n)	HC (n)	IBS Criteria	Zonulin Assay Kit	Units	Main Finding (IBS vs. HC)
Rezazadegan 2022 [41]	Iran	Case–control	IBS-D (61)	61	Rome IV	Zellbio	ng/mL	No significant difference (*p* = 0.888)
Singh 2019 [42]	USA	Case–control	IBS-D (50), IBS-C (50)	42	Rome III	ALPCO	ng/mL	Significantly higher in both IBS-D and IBS-C (*p* = 0.009 and 0.006)
Talley 2020 [43] ^1^	Australia/Sweden	Population- and clinic-based	IBS, mixed (40 population; 46 clinic)	182 (population)	Rome III	Not reported	pg/mg protein	No significant difference (population *p* = 0.5; clinic *p* = 0.7)
Barbaro 2020 [44]	Italy	Multicentre case–control	IBS-D (59)	25	Rome III	Cusabio	pg/mg protein	No significant difference (*p* = 1.000)
Kushlinskii 2022 [45] ^2^	Russia	Case–control	IBS, combined with IBD (29)	50	NR	Immundiagnostik	ng/mL	Significantly higher in mixed pathological group (*p* < 0.0002)
Linsalata 2018 [46]	Italy	Case–control	IBS-D (39)	20	Rome III	Immundiagnostik	ng/mL	No significant difference (IBS vs. HC)
Meira-de-Faria 2021 [47] ^3^	Sweden	Case–control	IBS-D (18), IBS-C (17), IBS-M (43)	40	Rome III	Cusabio/Bio-Rad mAb	ng/mL	Significantly higher with Cusabio (*p* < 0.001); significantly lower pre-HP2 with specific mAb (*p* < 0.01)
Peruhova 2022 [48]	Bulgaria	Case–control	IBS, subtype NR (15)	8	Rome IV	Cusabio	ng/mL	Significantly higher (*p* = 0.0315)

NR, not reported; mAb, monoclonal antibody. ^1^ The ELISA kit used by Talley et al. [43] was not specified in the original publication. The clinic-based cohort of Talley et al. [43] used patients with organic gastrointestinal diseases as the comparator rather than healthy controls. Only the population-based cohort (IBS vs. healthy controls) contributed to the primary synthesis; ^2^ Kushlinskii et al. [45] is retained as supportive, non-primary evidence. The study combined IBS patients with IBD patients in a single pathological group and did not report IBS-specific summary statistics; the diagnostic criteria for IBS were not specified (“NR”). This study did not contribute to the primary IBS-versus-control synthesis. ^3^ Meira-de-Faria et al. [47] reported opposite results with two different assays applied to the same samples: the Cusabio ELISA showed higher zonulin in IBS, whereas the pre-HP2-specific monoclonal antibody showed lower pre-HP2 in IBS. The study is therefore not counted as unambiguously positive. Matrix: Zonulin was measured in serum in all studies except Meira-de-Faria et al. [47], which measured plasma. Serum and plasma are not analytically interchangeable, and concentrations should not be directly compared across studies or pooled. Matrix data for each study are also provided in Supplementary Table S2.

**Table 2:** Summary of independent validation studies of commercial zonulin ELISA kits.

Validation Study	Assay Name	Manufacturer	Immunogen	Claimed Target	Actual Target(s) Identified	Matrix Tested	Method of Validation
Scheffler 2018 [38]	IDK Zonulin ELISA	Immundiagnostik AG	GGVLVQPG peptide	Zonulin/pre-HP2	Properdin	Serum	Western blot, mass spectrometry, immunoprecipitation
Ajamian 2019 [39]	Human Zonulin ELISA Kit	Cusabio Biotech	GGVLVQPG peptide	Zonulin/pre-HP2	Complement C3, haptoglobin	Serum	Recombinant zonulin, HP phenotyping, commercial ELISAs

HP, haptoglobin.

**Table 3:** Risk of bias assessment of included case–control studies using the Newcastle–Ottawa Scale.

Study	Selection (Max 4)	Comparability (Max 2)	Exposure (Max 3)	Total (Max 9)	Risk of Bias
Rezazadegan 2022 [41]	4	2	2	8	Low
Singh 2019 [42]	4	1	2	7	Low
Talley 2020 [43]	4	2	3	9	Low
Barbaro 2020 [44]	3	1	2	7	Low
Kushlinskii 2022 [45] *	2	0	2	4	Intermediate
Linsalata 2018 [46]	4	1	2	7	Low
Meira-de-Faria 2021 [47]	4	2	2	8	Low
Peruhova 2022 [48]	3	0	2	5	Intermediate

Max, maximum. * Kushlinskii et al. [45] was assessed for completeness and retained as supportive evidence. Its combined IBS/IBD cohort was the principal source of selection bias.

## Data Availability

No new data were created or analysed in this study. Data sharing is not applicable to this article.

## Supplementary Materials

The following supporting information can be downloaded at: https://www.mdpi.com/article/10.3390/biomedicines14102354/s1. PRISMA 2020 checklist (Word); PRISMA 2020 for Abstracts checklist (Word); Table S1: All of the excluded studies with reasons for exclusion and DOIs; Table S2: Data extracted from each included study; Table S3: Assay-specific risk-of-bias assessment of included studies.

## Abbreviations

The following abbreviations are used in this manuscript:

BMI	Body mass index
C3	Complement component 3
CXCR3	C-X-C chemokine receptor type 3
ELISA	Enzyme-linked immunosorbent assay
FODMAP	Fermentable oligosaccharides, disaccharides, monosaccharides, and polyols
GRADE	Grading of Recommendations Assessment, Development and Evaluation
HP	Haptoglobin
HP1	Haptoglobin 1 allele
HP2	Haptoglobin 2 allele
HP1-1	Haptoglobin 1-1 genotype
HP2-1	Haptoglobin 2-1 genotype
HP2-2	Haptoglobin 2-2 genotype
IBD	Inflammatory bowel disease
IBS	Irritable bowel syndrome
IBS-C	Irritable bowel syndrome, constipation-predominant
IBS-D	Irritable bowel syndrome, diarrhoea-predominant
IBS-M	Irritable bowel syndrome, mixed
I-FABP	Intestinal fatty acid-binding protein
IP	Immunoprecipitation
IQR	Interquartile range
mAb	Monoclonal antibody
MyD88	Myeloid differentiation primary response 88
NCGS	Non-coeliac gluten sensitivity
NOS	Newcastle–Ottawa Scale
NR	Not reported
pre-HP2	Pre-haptoglobin-2
PRISMA	Preferred Reporting Items for Systematic Reviews and Meta-Analyses
SD	Standard deviation
ZO-1	Zonula occludens-1
