# Supplementary material for: Circulating Zonulin in Irritable Bowel Syndrome: A Systematic Review of Conflicting Evidence and the Critical Impact of Assay Specificity
Source: Biomedicines. 2026 Oct 16;14(10):2354. doi: 10.3390/biomedicines14102354 (PMC13702017; doi:10.3390/biomedicines14102354)
Supplement: Supplementary file 1 [file biomedicines-14-02354-s001.zip › Supplementary Table S1.pdf]

Supplementary Table S1. All of the excluded studies with reasons for exclusion and DOIs

First author and year	*Reason for exclusion*	*DOI*
Bianco 2026	No healthy control group	10.3389/fphys.2026.1912788
Molino 2026	No healthy control group	10.1002/mnfr.70451
Güemes-González 2026	Non-original study (review)	10.1016/j.arcmed.2025.103321
Hoseini 2025	Non-original study (narrative review)	10.1186/s12967-025-07492-1
Maimaris 2025	Non-original study (narrative review)	10.3390/ijms262211174
Russo 2025	No healthy control group	10.3389/fphys.2025.1695163
Wierzbicka 2025	Fecal zonulin instead of serum/plasma	10.3389/fnut.2025.1603011
Li 2025	No healthy control group	10.3389/fcimb.2025.1616066
Laterza 2025	No healthy control group	10.3390/nu17101708
Jafari Nasab 2025	Non-original study (protocol)	10.1136/bmjopen-2024-086521
Nasab 2025	No healthy control group	10.1186/s12937-025-01132-6
Bianco 2024	No healthy control group	10.3390/nu16234181
Linsalata 2024	Fecal zonulin instead of serum/plasma	10.3390/nu16162706
Gargari 2024	No healthy control group	10.1080/19490976.2023.2298246
Kovaleva 2023	No healthy control group	10.3390/jcm12186064

Gaus 2023	Fecal zonulin instead of serum/plasma	10.26442/00403660.2023.02.202111
Ibrahim 2022	Not IBS patients or no IBS-specific data	10.5599/admet.905
Ohlsson 2022	Non-original study (review)	10.3892/mmr.2022.12742
Kleppe 2022	Not IBS patients or no IBS-specific data	10.1002/eat.23678
Massier 2021	Non-original study (comment/editorial)	10.1136/gutjnl-2020-323026
Prospero 2021a	No healthy control group	10.3390/nu13072469
Prospero 2021b	No healthy control group	10.1186/s12876-021-01820-7
Linsalata 2021	Fecal zonulin instead of serum/plasma	10.3390/nu13031011
Petkov 2021	No healthy control group	Not reported; PMID: 33930939
Caviglia 2020a	No healthy control group	10.3390/jcm9082353
Caviglia 2020b	No healthy control group	10.3390/jcm9030800
Vojdani 2020	Not IBS patients or no IBS-specific data	10.3390/ijms21041381
Leech 2019	Non-original study (systematic review)	10.1111/ijcp.13385
Moser 2019	Fecal zonulin instead of serum/plasma	10.1007/s00394-018-1826-7
Vojdani 2019	Not IBS patients or no IBS-specific data	10.3748/wjg.v25.i9.1050
Simeonova 2018	Non-original study (review)	10.2174/1568026618666181115100610
Ohlsson 2017	Not IBS patients or no IBS-specific data	10.3390/ijms18030582
