# Supplementary material for: Circulating Zonulin in Irritable Bowel Syndrome: A Systematic Review of Conflicting Evidence and the Critical Impact of Assay Specificity
Source: Biomedicines. 2026 Oct 16;14(10):2354. doi: 10.3390/biomedicines14102354 (PMC13702017; doi:10.3390/biomedicines14102354)
Supplement: Supplementary file 1 [file biomedicines-14-02354-s001.zip › Supplementary Table S2.pdf]

Table S2. Data extracted from each included study.

Data item	*Linsalata 2018*	*Singh 2019*	*Talley 2020*	*Barbaro 2020*	*Kushlinskii 2022*	*Meira-de-Faria 2021*	*Peruhova 2022*	*Rezazadegan 2022*
**Country**	Italy	USA	Australia/Sweden	Italy	Russia	Sweden	Bulgaria	Iran
**Design**	Case-control	Case-control	Population + clinic	Multicentre case-control	Case-control	Case-control	Case-control	Case-control
**Setting**	Tertiary	Tertiary	Population + tertiary	Multicentre	Tertiary	Tertiary	Tertiary	Tertiary
**IBS n**	39	100	40 (pop), 46 (clinic)	59	29	78	15	61
**Control n**	20	42	182 (pop), 26 (disease)	25	50	40	8	61
**Age (IBS)**	40.1	32.1 (D), 31.6 (C)	62.1 (pop), 45.0 (clinic)	43.5	41 (median)	30 (median)	36 ± 10	37.0 ± 8.5
**Age (controls)**	39.7	35.2	—	33.0	42 (median)	32 (median)	NR	37.3 ± 9.0
**% female (IBS)**	84.6%	24% (D), 96% (C)	45.9% (pop), 67.4% (clinic)	52.5%	65.5%	88.5%	80%	62.3%
**% female (controls)**	65%	78.6%	—	64%	54%	90%	NR	62.3%
**IBS subtype**	IBS-D	IBS-D, IBS-C	Mixed	IBS-D	Mixed	IBS-D, IBS-C, IBS-M	NR	IBS-D
**Diagnostic criteria**	Rome III	Rome III	Rome III	Rome III	NR	Rome III	Rome IV	Rome IV
**ELISA kit**	Immundiagnostik	ALPCO	NR	Cusabio	Immundiagnostik	Cusabio + Bio-Rad mAb	Cusabio	Zellbio
**Matrix**	Serum	Serum	Serum	Serum	Serum	Plasma	Serum	Serum

**Reporting units**	ng/mL	ng/mL	pg/mg	pg/mg	ng/mL	ng/mL	ng/mL	ng/mL
**Zonulin (IBS)**	~70 (Fig. 3A exact values not reported)	65.8 (D), 67.2 (C)	3999.8 (pop), 3577.9 (clinic)	7.9 ± 10.7	49.7 (median)	3,51 (median) [Cusabio], Pre-HP2: 19,6 (median) [mAb]	0.378 ± 0.13	15.06 ± 3.86
**Zonulin (controls)**	~54 (Fig. 3A exact values not reported)	45.2	3755.1 (pop), 3407.2 (disease)	5.5 ± 5.5	38.3 (median)	2,47 (median) [Cusabio], Pre-HP2: 50,63 (median) [mAb]	0.250 ± 0.14	14.96 ± 3.71
**p-value**	NS	0.009 (D), 0.006 (C)	0.5 (pop), 0.7 (clinic)	NS	< 0.0002	< 0.001 (Cusabio); < 0.01 (mAb)	0.0315	0.888
**Correlation**	None	r = 0.45 stool freq; r = 0.43 dissatisfaction	None	None	None	None	None	r = −0.061 (IBS-SSS); NS
**NOS score**	7	7	9	7	4	8	5	8

NR, not reported; NS, not significant; NOS, Newcastle-Ottawa Scale; mAb, monoclonal antibody; D, diarrhoea-predominant; C, constipation-predominant.

Values are presented as mean (range) unless otherwise indicated. Linsalata et al. reported serum zonulin only graphically; exact numerical values are not available in the published article. Meira-de-Faria et al. reported zonulin concentrations measured with two different assays (Cusabio ELISA and a monoclonal pre-HP2 antibody), which produced opposite results; both values are presented for completeness. mAb, monoclonal antibody; HC, healthy controls; IBS, irritable bowel syndrome.
In the Meira-de-Faria study, "zonulin" refers to the measurement obtained with the Cusabio ELISA kit, and "pre-HP2" refers to the measurement obtained with a monoclonal antibody specific for pre-haptoglobin-2. The two measurements did not agree, which is the central finding of the study.
