# Supplementary material for: Circulating Zonulin in Irritable Bowel Syndrome: A Systematic Review of Conflicting Evidence and the Critical Impact of Assay Specificity
Source: Biomedicines. 2026 Oct 16;14(10):2354. doi: 10.3390/biomedicines14102354 (PMC13702017; doi:10.3390/biomedicines14102354)
Supplement: Supplementary file 1 [file biomedicines-14-02354-s001.zip › Supplementary Table S3.pdf]

Table S3. Assay-specific risk-of-bias assessment of included studies.

Study	1. Independent assay target validation	2. Haptoglobin genotyping	3. Biological matrix specified	4. Sample handling reported	5. Assay details reported	6. Blinding of laboratory personnel	7. Batch effects addressed	Overall assay-specific concern
**Linsalata 2018**	High (Immundiagnostik; detects properdin)	Not reported	Low (serum)	Low (−80 °C)	Unclear (kit named)	Not reported	Not reported	**High**
**Singh 2019**	High (ALPCO; not validated)	Not reported	Low (serum)	Unclear (repository)	Low (kit + catalogue)	Not reported	Not reported	**High**
**Talley 2020**	High (kit not reported)	Not reported	Low (serum)	Unclear	High (kit not reported)	Not reported	Not reported	**High**
**Barbaro 2020**	High (Cusabio; detects C3/haptoglobin)	Not reported	Low (serum)	Low (−20 °C)	Low (kit + range + sensitivity)	Low (blinded analysis)	Not reported	**High**
**Kushlinskii 2022**	High (Immundiagnostik; detects properdin)	Not reported	Low (serum)	Not reported	Unclear (kit + sensitivity)	Not reported	Not reported	**High**
**Meira-de-Faria 2021**	**Low** (Bio-Rad mAb validated)	**Yes** (HP1/HP2 PCR)	**Low** (plasma)	**Low** (−80 °C)	**Low** (both assays detailed)	Not reported	Not reported	**Low**
**Peruhova 2022**	High (Cusabio; detects C3/haptoglobin)	Not reported	Low (serum)	Not reported	Low (kit + range + sensitivity)	Not reported	Not reported	**High**
**Rezazadegan 2022**	High (Zellbio; not validated)	Not reported	Low (serum)	Low (−80 °C)	Unclear (kit named)	Not reported	Not reported	**High**

Rating scale: Low concern / Unclear / High concern. C3, complement component 3; mAb, monoclonal antibody; HP, haptoglobin; PCR, polymerase chain reaction.

Legend for the assessment items

#	Item	What it assesses
1	Independent assay target validation	Whether the assay has been independently shown to detect its claimed target
2	Haptoglobin genotyping	Whether participants were genotyped for HP1/HP2 alleles (essential because pre-HP2 cannot be produced by HP1-1 individuals)
3	Biological matrix specified	Whether serum or plasma was used and clearly stated
4	Sample handling reported	Whether storage temperature and processing conditions were reported
5	Assay details reported	Whether kit name, catalogue number, detection range, and sensitivity were provided
6	Blinding of laboratory personnel	Whether laboratory staff performing the assay were blinded to group allocation
7	Batch effects addressed	Whether samples were run in batches or inter-batch variability was controlled
